# Differentially-Charged Liposomes Interact with Alphaherpesviruses and Interfere with Virus Entry

**DOI:** 10.3390/pathogens9050359

**Published:** 2020-05-08

**Authors:** Oleksandr Kolyvushko, Juliane Latzke, Ismail Dahmani, Nikolaus Osterrieder, Salvatore Chiantia, Walid Azab

**Affiliations:** 1 Institut für Virologie, Robert von Ostertag-Haus, Zentrum für Infektionsmedizin, Freie Universität Berlin, Robert-von-Ostertag-Street 7-13, 14163 Berlin, Germany; olek@zedat.fu-berlin.de (O.K.); j.latzke@fu-berlin.de (J.L.); no.34@fu-berlin.de (N.O.); 2 Institute of Biochemistry and Biology, University of Potsdam, Karl-Liebknecht-Street 24-25, 14476 Potsdam, Germany; dahsmail@gmail.com (I.D.); chiantia@uni-potsdam.de (S.C.)

**Keywords:** alphaherpesvirus, EHV-1, phosphatidylserine, inhibition, pathogen host interaction

## Abstract

Exposure of phosphatidylserine (PS) in the outer leaflet of the plasma membrane is induced by infection with several members of the *Alphaherpesvirinae* subfamily. There is evidence that PS is used by the equine herpesvirus type 1 (EHV-1) during entry, but the exact role of PS and other phospholipids in the entry process remains unknown. Here, we investigated the interaction of differently charged phospholipids with virus particles and determined their influence on infection. Our data show that liposomes containing negatively charged PS or positively charged DOTAP (N-[1-(2,3-Dioleoyloxy)propyl]-N,N,N-trimethylammonium) inhibited EHV-1 infection, while neutral phosphatidylcholine (PC) had no effect. Inhibition of infection with PS was transient, decreased with time, and was dose dependent. Our findings indicate that both cationic and anionic phospholipids can interact with the virus and reduce infectivity, while, presumably, acting through different mechanisms. Charged phospholipids were found to have antiviral effects and may be used to inhibit EHV-1 infection.

## 1. Importance

Cell entry is one of the first steps in virus infection of the target cell. Equid herpesvirus type 1 (EHV-1) is an important pathogen affecting equids worldwide. EHV-1 binding to cellular receptors involves activation of phospholipid scramblase and subsequent exposure of the phosphatidylserine (PS) on the outer leaflet of the plasma membrane. Application of phospholipids can change the outcome of infection, and understanding the mechanism and factors involved in this step may lead to improved control of infection.

## 2. Introduction

Phospholipids have been shown to be necessary for enveloped viruses to promote infection. Phosphatidylserine (PS) is used as a receptor by different viruses [1,2]. Recently, it was shown that PS exposure on cell surface occurs shortly after equid herpesvirus type 1 (EHV-1) contacts the cell [3]. Similar findings for another alphaherpesvirus, herpes simplex virus type 1 (HSV-1), were reported [4]. Exogenous PS and the anionic lipid phosphatidylglycerol facilitate cell-to-cell fusion of cells expressing human immunodeficiency virus 1 (HIV-1) proteins. This led to the conclusion that specific interactions can occur between virus particles and cellular phospholipids [5]. Here, we investigated the role of PS as well as other phospholipids during virus infection. Furthermore, we assessed the specificity of the interaction between EHV-1 and differentially charged phospholipids. To this end, we used small unilamellar vesicles (SUV) composed of exogenous lipids that have different charges: (i) negatively charged PS, (ii) positively charged DOTAP (N-[1-(2,3-Dioleoyloxy)propyl]-N,N,N-trimethylammonium), or (iii) neutral phosphatidylcholine (PC). We investigated the interaction between virus particles and the different phospholipids by means of surface plasmon resonance (SPR) and visualized virus interaction with giant unilamellar vesicles (GUV) or large unilamellar vesicles (LUV) via confocal microscopy.

## 3. Results

### 3.1. DOTAP and PS Liposomes Inhibit Viral Infection

**Figure 1:**
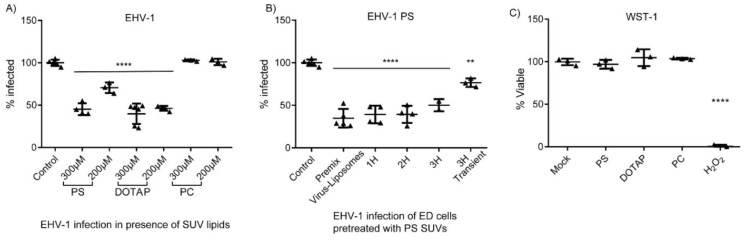
Phospholipids inhibit equid herpesvirus type 1 (EHV-1) infection. (**A**) Effects of lipid concentration on EHV-1 infection. Equine dermal (ED) cells were treated with 200 μM or 300 μM of small unilamellar vesicles (SUVs) (phosphatidylserine (PS); N-[1-(2,3-Dioleoyloxy) propyl]-N,N,N-trimethylammonium (DOTAP):phosphatidylcholine (PC) 1:1; or PC SUVs) for 3 h and infected with EHV-1 at an MOI of 0.1. GFP expression at 24 h post infection was measured by FACS. (**B**) ED cells were treated with 300 μM of PS SUVs for different times (0; Premix Virus-Liposomes, 1, 2, or 3 h) and then infected with EHV-1 at MOI of 0.1. GFP expression at 24 h post infection was measured by FACS. Control: cells infected with viruses without previous treatment with lipids. 3H Transient: SUVs were removed by washing the cells three times with PBS before infection. (**C**) Cell viability assay, cells were treated with SUVs composed of PS, DOTAP:PC (1:1), or PC lipids for 24 h. Mock-treated cells and cells treated with H_2_O_2_ (30%) were used as the 100% and 0% viability controls, respectively. No significant differences between treatment groups and mock control were found. *p* < 0.05, Kolmogorov–Smirnov normality test followed by one-way ANOVA with Dunnett’s multiple comparison test. Asterisks indicate a significant difference of lipid-treated to the control non-treated cells.

Three types of lipids were tested: positively charged DOTAP, negatively charged PS, and neutral PC. Equine dermal (ED) cells were treated with SUVs containing PS, PC, or DOTAP:PC (1:1), at a concentration of 200 μM or 300 μM for 3 h prior to infection with EHV-1. ED cells infected without previous SUV treatment were used as a control. Positively and negatively charged DOTAP and PS lipids significantly inhibited EHV-1 infection (Figure 1A). In contrast, neutral PC SUVs had no effect on virus infection. At a concentration of 300 μM of all lipid preparations, none of the SUVs significantly affected cell viability after a 24 h incubation period (Figure 1C). 

In another experiment, ED cells were incubated with 300 μM PS SUVs for different times (0, 1, 2, or 3 h) and then infected with EHV-1 for 24 h. At all time points, virus infection was significantly inhibited in the presence of PS (Figure 1B). The dataset termed “3H transient” represents the treatment of ED cells with 300 μM PS SUVs for 3 h, before the SUVs were removed by 3 consecutive washes with PBS and addition of EHV-1 to the cells at an MOI of 0.1. Interestingly, there was no inhibition of virus infection in the absence of PS, indicating that the inhibitory effect of PS is transient and only effective if PS liposomes are present at the time of virus exposure (Figure 1B). Longer incubation of cells with PS rescued EHV-1 infectivity, although at significantly lower levels when compared with that in non-treated cells (Figure 1B). It is worth mentioning that EHV-4, a close relative to EHV-1, was also inhibited in a similar fashion (data not shown). 

### 3.2. Interaction of Viral Particles with Phospholipids

**Figure 2:**
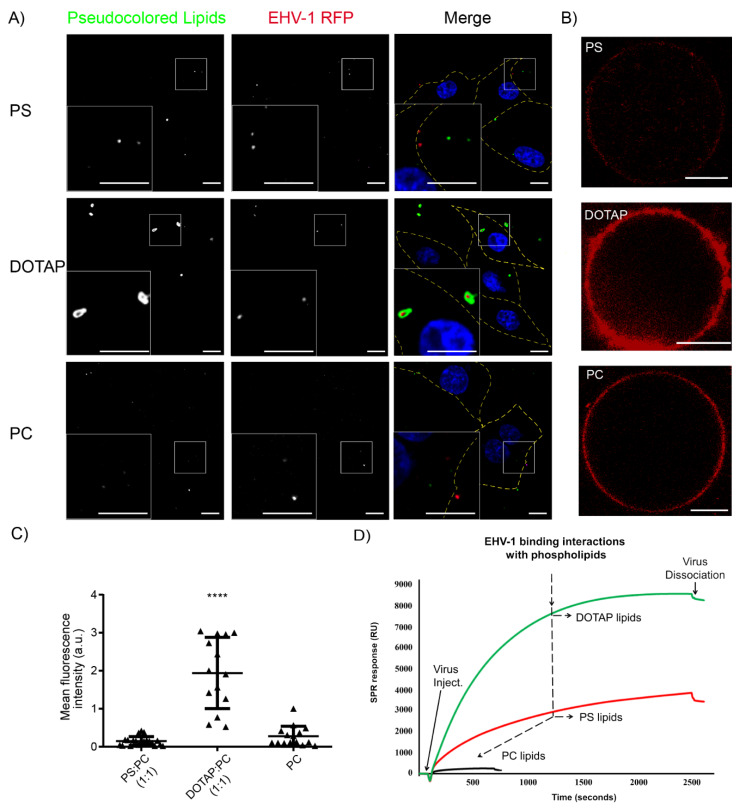
Virus interaction with phospholipids. (**A**) EHV-1-RFP (MOI = 5) was mixed with fluorescently labeled large unilamellar vesicles (LUVs) of PS, PC or DOTAP:PC (1:1). The mixture was applied to ED cells that were incubated for 1 h on ice. Green: pseudocolored fluorescently labeled LUVs; Red: RFP-labeled viruses; Blue: DAPI stained nucleus. Merge panels: dotted lines represent the boundaries of cells. Scale bar = 10 μm. (**B**) Binding of EHV-1-RFP to giant unilamellar vesicles (GUVs). Pictures of GUVs made of PS:PC (1:1), DOTAP:PC (1:1), and PC with bound RFP-labeled virus. Scale bars indicate 10 µm. (**C**) Quantification of the average signal in 15–21 GUVs from two independent experiments for each lipid compositions: PC, PC:PS (1:1), or DOTAP:PC (1:1). **** *p* < 0.0001 (one-way ANOVA with Dunnett’s test was used to correct for multiple comparisons). (**D**) Surface plasmon resonance (SPR) analysis of EHV-1 interactions with model membrane composed of neutral, cationic, or anionic phospholipids. Representative sensorgrams after curve alignment show the virus association to the immobilized lipid monolayer composed of DOTAP 100 mol%, PC 100 mol%, or PS 100 mol%. RU: corrected SPR response unit.

Fluorescently (Cy 5.5 PE)-labeled LUVs composed of three different lipids (PS, PC, or DOTAP:PC 1:1) were mixed with EHV-1-RFP at an MOI of 5 and added to ED cells. The cells were incubated for 1 h on ice to allow virus binding without internalization. Cells were fixed with 4% PFA and visualized with a confocal microscope. Viral particles (all of the 103 virus particles counted blindly on 10 cells) were found to significantly colocalize with DOTAP LUVs when compared to PS LUVs (*p* value ≤ 0.00001; Fisher’s exact test). Colocalization was much less frequently observed with PS (27 out 105 virus particles counted blindly in 13 cells) or PC (4 out of 108 virus particles counted blindly in 17 cells) LUVs (Figure 2A).

Under cell-free conditions, fluorescent confocal microscopy was used to quantify the specific binding between RFP-labeled EHV-1 and GUVs of different composition: PC, PC:PS (1:1), or DOTAP:PC (1:1) (Figure 2B); GUVs composed entirely of DOTAP or PS could not be obtained at high enough yield. Mean fluorescence intensity was measured in 15–21 vesicles for each lipid composition in two independent experiments. Our data showed that EHV-1 bound more tightly to DOTAP:PC (1:1) GUVs when compared to PS:PC (1:1) and PC GUVs (Figure 2B,C). Additionally, surface plasmon resonance (SPR) was used to detect binding dynamics of viral particles to PS, PC, or DOTAP lipids (Figure 2D). A lipid monolayer was formed, and purified virus was injected until an equilibrium was reached. In the case of PC, binding was very low (ca. 400 ± 100 RU, n = 2) and equilibrium was reached after around 10 min. Binding of the virus to PS monolayers was intermediate (ca. 3500 ± 800 RU, n = 4), but should only be considered an estimate, since no clear equilibrium was observed even after 40 min. Binding to DOTAP reached an equilibrium within the injection time and was quantified at approximately 7000 ± 500 RU (n = 2). 

## 4. Discussion

We report here that PS and DOTAP inhibit the infection of ED cells by EHV-1. The inhibition is immediate, reversible, and dose-dependent. Previously, we showed that EHV-1 facilitates scramblase-dependent exposure of PS on the outer leaflet of the plasma membrane, suggesting that there is a specific, yet unknown, role for PS in fusion with the plasma membrane [3]. Thus, we surmised that integration of external PS would promote fusion and enhance the infection process [5]. Contrary to our expectations, we found that EHV-1 infection was reduced in the presence of PS.

The hypothesis that PS liposomes are blocking the infection after integration into the plasma membrane of the target cell, as was described for HIV before [5], was not supported by our data, because (i) there was no significant change in virus inhibition with increase in duration of incubation times, (ii) significant inhibition was observed immediately after addition (0 h time point), and (iii) removal of PS liposomes prior to infection rescued virus infection. From our results we concluded that the mechanism of virus entry, although appearing similar, is distinct between HIV and herpesviruses. 

Higher concentration of PS resulted in more pronounced reduction of infection. EHV-1 induces localized PS exposure during the early stages of infection [3] and, presumably, viral particles have the capacity to bind to PS to facilitate membrane fusion. Possibly, addition of external PS may transiently bind to the viral particles and thus reduce infectivity. When media containing PS was removed, cells regained susceptibility to infection independently of previous exposure to PS. We surmise that free PS liposomes in media are interfering specifically with the process of virus–cell interaction (binding) with EHV-1. It is likely that EHV-1 envelope glycoproteins, like those of other viruses, feature a phospholipid (PS)-binding domain that enables virus binding to different phospholipids of the plasma membrane to facilitate entry [3,6,7]. Addition of exogenous PS can interact with herpesviral glycoproteins, particularly gH/gL and gB, and block virus entry as was described for other viruses [6]. On the other hand, it is possible that PS is redistributing the charges on the cell surface in a fashion that makes the initial contact between virus particles and cell surface proteoglycans containing heparan sulfate less likely, thus reducing the probability of virus entry. However, the exact mechanism of how PS is blocking the infection needs to be investigated. 

Our confocal microscopy and SPR data both confirmed that PS interacts with EHV-1. Although the microscopy data did not show strong interaction between PS and virus particles, the SPR data indicated a stronger interaction as compared to neutral liposomes (PC). It became clear in our study that SPR analysis is more sensitive than microscopic examination. It can detect binding of small amounts of phospholipids from exosomes, cell debris, or other impurities from the virus purification process that could interact with virus particles, but remain undetected by confocal microscopy. This approach is complementary to confocal microscopy, which is more specific since it relies on labeled viral and lipid particles. 

Our data provides evidence of virus interaction with DOTAP, as is the case with PS. Both the SPR and confocal microscopy results showed that interaction of EHV-1 with the cationic DOTAP is stronger than that with PS, whereas there is no interaction with PC. Positively charged DOTAP also had an inhibitory effect on EHV-1 infection. However, unlike in the case of PS lipids, the strong interaction between DOTAP and virus particles (Figure 2) can be explained by the negative charge of the virus particle that can attract cationic DOTAP vesicles. The strong liposome–virus interaction in turn leads to a reduction of infection through preventing virus from interacting with its cognate cellular receptors. The electrostatic interaction (of DOTAP with EHV-1) is in-line with the data describing that herpesviruses possess a negative overall charge [8], probably due to anionic lipids present in the envelope and to the protein’s net charges and glycans, which carry charged carboxy- or sulfate groups. Zwitterionic lipid PC, on the other hand, showed no binding with EHV-1, and had no effect on infection. 

Strong inhibitory effect of DOTAP and PS that was observed in vitro, could point further investigation into the biophysical interaction between virus and the cell and would improve our understanding of the infection process. Furthermore, as phospholipids are important for the entry of several viruses, they might have a great potential as antiviral agents. 

## 5. Materials and Methods

### 5.1. Viruses and Cells

Equid herpesvirus type 1 strain RacL11 EHV-1-RFP [9] with a red fluorescent protein (RFP) fused to the small capsid protein VP26 [3] was used in this study. The virus further expresses the enhanced green fluorescent protein (eGFP) for efficient identification of infected cells. The virus was grown on primary equine dermal (ED) cells (CCLV-RIE 1222, Federal Research Institute for Animal Health, Germany) as described before [3]. The cells were propagated in Isocove’s Liquid Medium with stable glutamine (Pan-Biotech GmbH) supplemented with 20% fetal calf serum (Pan-Biotech GmbH), 0.5% penicillin (Roth), 0.5% streptomycin (Alfa Aesar), 1% sodium pyruvate 100 mM (Pan-Biotech GmbH), and 1% nonessential amino acids (Merck KGaA). For microscopy experiments, virus was purified by ultracentrifugation over a 30% sucrose solution followed by sucrose step gradient ultracentrifugation exactly as described before [10]. 

### 5.2. Phospholipids

Phosphatidylserine (PS; 1,2-dioleoyl-sn-glycero-3-phospho-L-serine; number: 840035), phosphatidylcholine (PC; 1,2-dioleoyl-sn-glycero-3- phosphocholine; number: 850375), and DOTAP (1,2-dioleoyl-3-trimethylammonium-propane; number: 890890) were purchased from Avanti Polar Lipids, USA. Multilamellar vesicles (MLVs) were prepared at concentration of 1 mM. Lipid stock was dissolved in ultrapure chloroform (Sigma Aldrich, Germany) in a glass vial. The solvent was evaporated under gentle flow of nitrogen and lipids were kept in a desiccator with calcium chloride overnight. The dry lipid film was resuspended in phosphate-buffered saline (PBS) with at least 2 h of shaking at 200 rpm at room temperature. The resulting opaque 1 mM MLV solution was stored at –80 °C. Large unilamellar vesicles (LUVs) were generated via extrusion through a polycarbonate membrane with 0.1 μm pore size. The extruder setup consisted of two syringes and the extruder itself, all held together by a holding block (Avanti Polar Lipids, USA). Extrusion was performed at a constant temperature of 37 °C, and LUVs were stored at 4 °C for up to 3 days. The mixture of MLVs was passed through the membrane 31 times to ensure homogeneous LUV size. Small unilamellar vesicles (SUVs) were produced through ultrasonication of MLVs in 1.5 mL tubes at room temperature, with ultrasonic frequency: 35 kHz, ultrasonic peak output: 320 W, in a Sonorex Super RK100 (Bandelin, Germany). Ultrasonication was carried out in two 15-min steps, with vortexing in between. SUVs and LUVs used throughout this study were composed of either 100 mol% PS, 100 mol% PC, or 1:1 DOTAP:PC. All SUVs and LUVs were labeled via addition of DHPE, Oregon Green™ 488 (1,2-Dihexadecanoyl-sn-Glycero-3-Phosphoethanolamine, Thermo Fisher Scientific, USA-IL, Rockfeld) and 18:0 Cy5.5 PE1,2-distearoyl-sn-glycero-3-phosphoethanolamine-N-(Cyanine 5.5) (Avanti Polar Lipids, USA). During the preparation process, a final concentration of 0.01 to 0.05 mol% was used for both fluorescent lipids. All lipid preparations were tested for their possible cytotoxic effects on cells using WST-1 cell proliferation assay kit (Cayman chemical, USA) [11,12]. 

Giant unilamellar vesicles (GUVs) were produced through electroformation [13]. Briefly, a lipid solution in organic solvent with the desired lipid components was dried on indium tin oxide (ITO) coated glass slides. After evaporation of the solvent, a swelling solution of 150 mM sucrose was carefully added. The two ITO slides were separated by a 3-mm thick Teflon spacer. An alternating current (AC) with a 10 Hz sine wave, and electric potential difference of 0.5 V, was applied for the first 30 min, following by 1.0 V for 30 min and 1.6 V for 30 min. The resulting GUVs were assessed via confocal microscopy. In order to increase the yield of the electroformation process, PS-containing GUVs were prepared with a composition of 1:1 PS:PC (rather than 100% PS, as used for SUVs or LUVs).

### 5.3. Flow Cytometry

Approximately 30,000 cells were treated with freshly prepared SUVs of the appropriate lipids (PS, PC, or DOTAP:PC 1:1; 300 µM), and incubated for different periods (0, 1, 2, and 3 h) at 37 °C and 5% CO_2_. Afterwards, cells were infected in the presence of SUVs with EHV-1 at a multiplicity of infection (MOI) of 0.1. At the 0 time point (Premix Virus-Liposomes), lipids were mixed with viruses and immediately added to the cells. Mock-treated cells (i.e. cells without addition of lipids) were infected with EHV-1 and used as positive controls. In another experiment, cells were incubated with different concentrations, 200 µM or 300 µM, of lipids (PS, PC, or DOTAP:PC 1:1) for 3 h before infection with EHV-1 (MOI = 0.1). Twenty-four hours post infection, 10,000 cells were analyzed using CytoFLEX S (Beckman Coulter, Germany). The subset of GFP positive cells among viable population was considered infected. The percent of GFP-positive cells in the mock-treated group was set to 100%. 

### 5.4. Surface Plasmon Resonance

This experiment was performed on a SPR GE Biacore J Biomolecular Interaction Analyser instrument (Uppsala, Sweden) using a lipid-coated HPP sensor chip (XanTec bioanalytics GmbH) prepared according to the protocol provided by the chip vendor. All solutions were freshly prepared, degassed, and filtered through 0.22 μm-pore filters and measurements were performed at 24 °C in PBS (pH 7.4). Prior to use, the surface of the HPP chip was cleaned by an injection of the nonionic detergent *N*-octyl β-d-glucopyranoside (100 μL, 40 mM) at low flow setting. PS (100 mol%), PC (100 mol%), and DOTAP (100 mol%) liposomes (1 mM) were injected as required over the surface of the HHP sensor chip for 60 min. Note that DOTAP and PS liposomes did not contain PC, for all SPR experiments, in order to avoid uncertainties regarding the actual final composition of the lipid monolayer. One of the two available chip channels (flow cells) was incubated in all experiment with PS vesicles. The second channel was treated with either PC or DOTAP vesicles. Then, NaOH (200 μL, 20 mM) was injected several times until producing a stable baseline with a signal ranging from 2500 to 3200 resonance units (RU), compatible with the presence of a stable lipid monolayer in both channels. EHV-1 was injected simultaneously over the lipid monolayers in both channels and virus binding was monitored for up to 40 min, to provide sufficient time for the association phase to reach saturation equilibrium levels (R_eq_) [14]. The dissociation phase was monitored for 5 min. Each lipid composition was analyzed in independent duplicates (n = 2 for DOTAP and PC, n = 4 for PS).

### 5.5. Microscopy

Confocal laser scanning microscopy was conducted with Nikon Eclipse Ti Visitron microscope (Visitron Systems GmbH, Germany) to visualize the colocalization of LUVs with RFP-labeled viruses. A 100× oil immersion objective was used in combination with an EMCCD camera and the VisiVIEW imaging software (Visitron Systems GmbH, Germany). Twenty-four hours before the experiment, 2000 ED cells were plated in a µ-Slide 8-well (ibidi, USA). On the day of experiment, cells were incubated with a mixture of EHV-1-RFP (MOI of 5) and fluorescently labeled LUVs of PS, PC, or DOTAP:PC 1:1 (300 µM) for 1 h on ice. Cells were fixed with 4% paraformaldehyde (PFA), visualized under the microscope, and virus particles (RFP-labeled) were counted as colocalized if the visible portion of the point spread function of the signals were overlapping. The experiment was conducted blindly three independent times. 

GUVs were observed with a Zeiss LSM780 system (Carl Zeiss, Oberkochen, Germany) using a 40×, 1.2 numerical aperture water-immersion objective. A fresh suspension of GUVs (either PS:PC 1:1, PC, or DOTAP:PC 1:1) was released from the ITO slide and transferred to imaging chambers. EHV-1-RFP virus was added to allow the interaction with the GUVs. The mixture was incubated at room temperature for at least 20 min prior to imaging. Mean fluorescence intensities values corresponding to the EHV-1-RFP bound to the GUVs membrane were quantified using Zen Black Software (Carl Zeiss). The analyzed regions were selected manually by drawing two concentric circles delimiting the GUV membrane.

### 5.6. Statistical Analysis

Experiments were performed in triplicate, unless specified otherwise. Image analysis was performed in a blinded fashion. One-way ANOVA was performed using GraphPad Prism (GraphPad Software, San Diego, CA, USA). The mean of each treatment groups was compared to the mean of the control group. The Dunnet’s test was used to correct for multiple comparisons. Fisher’s exact test was used to compare colocalization of virus particles with DOTAP compared to PS. Values of *p* < 0.05 are considered significant.
